# Data-driven optimization for microgrid control under distributed energy resource variability

**DOI:** 10.1038/s41598-024-58767-4

**Published:** 2024-05-11

**Authors:** Akhilesh Mathur, Ruchi Kumari, V. P. Meena, V. P. Singh, Ahmad Taher Azar, Ibrahim A. Hameed

**Affiliations:** 1 Department of Electrical Engineering, Malaviya National Institute of Technology, Jaipur, Rajasthan 302017 India; 2 Department of Electrical and Electronics Engineering, Amrita School of Engineering, Amrita Vishwa Vidyapeetham, Bengaluru, India; 3 College of Computer and Information Sciences, Prince Sultan University, 11586 Riyadh, Saudi Arabia; 4 Automated Systems and Soft Computing Lab (ASSCL), Prince Sultan University, Riyadh, Saudi Arabia; 5 Faculty of Computers and Artificial Intelligence, Benha University, Benha, 13518 Egypt; 6 Department of ICT and Natural Sciences, Norwegian University of Science and Technology, Larsgardsvegen, 2, 6009 Alesund, Norway

**Keywords:** Microgrids, Stochastic process, Optimal scheduling, Monte Carlo simulation, K-mean clustering, Probability distribution function, Grey-Wolf optimization, Jaya algorithm, Energy science and technology, Engineering

## Abstract

The integration of renewable energy resources into the smart grids improves the system resilience, provide sustainable demand-generation balance, and produces clean electricity with minimal leakage currents. However, the renewable sources are intermittent in nature. Therefore, it is necessary to develop scheduling strategy to optimise hybrid PV-wind-controllable distributed generator based Microgrids in grid-connected and stand-alone modes of operation. In this manuscript, a priority-based cost optimization function is developed to show the relative significance of one cost component over another for the optimal operation of the Microgrid. The uncertainties associated with various intermittent parameters in Microgrid have also been introduced in the proposed scheduling methodology. The objective function includes the operating cost of CDGs, the emission cost associated with CDGs, the battery cost, the cost of grid energy exchange, and the cost associated with load shedding. A penalty function is also incorporated in the cost function for violations of any constraints. Multiple scenarios are generated using Monte Carlo simulation to model uncertain parameters of Microgrid (MG). These scenarios consist of the worst as well as the best possible cases, reflecting the microgrid’s real-time operation. Furthermore, these scenarios are reduced by using a k-means clustering algorithm. The reduced procedures for uncertain parameters will be used to obtain the minimum cost of MG with the help of an optimisation algorithm. In this work, a meta-heuristic approach, grey wolf optimisation (GWO), is used to minimize the developed cost optimisation function of MG. The standard LV Microgrid CIGRE test network is used to validate the proposed methodology. Results are obtained for different cases by considering different priorities to the sub-objectives using GWO algorithm. The obtained results are compared with the results of Jaya and PSO (particle swarm optimization) algorithms to validate the efficacy of the GWO method for the proposed optimization problem.

## Introduction

Microgrid (MG) is a scaled-down version of the conventional grid. It is self-sufficient and can supply the local demands of a particular geographic area. The active components of the MG are renewable energy sources like wind turbines (WT), photovoltaic (PV), micro-hydro generators, biomasses, fuel cells, etc. The other associated components of MG are energy storage units, combined heat and power (CHP) units, thermal and electric loads, etc.^1^.

For ensuring supply reliability, fuel savings, lesser emissions, voltage security, full exploitation of renewable potential, and coordinated output of multiple DGs, there is a need for energy management and optimal dispatch of microgrids. Two different approaches that have widely been used in the literature for the optimal operation of MG are (a) deterministic approaches and (b) heuristic optimization approaches^2–8^.

Deterministic algorithms like linear programming, mixed-integer linear programming, and dynamic programming have been used in articles^9–15^ for unit commitment and economic load dispatch (ELD) of microgrids with or without the energy storage system. Various objectives, i.e. cost minimization, reliability maximization, emission reduction, power loss minimization, voltage security, and utilization of bio-waste in microgrids, are developed with multiple constraints in these papers. However, deterministic methods have some drawbacks: (a) they take more time with the complexity of the problem; (b) they become intractable with the increase in the number of parameters; (c) they have a high dependency on the initial solutions; (d) these methods are gradient-dependent, etc. Using meta-heuristic algorithms has resolved the issues related to deterministic methods. Some of the meta-heuristic algorithms, like a genetic algorithm (GA), modified genetic algorithm, particle swarm optimization (PSO), modified particle-swarm optimization (MPSO), grey-wolf optimization (GWO), artificial fish algorithms, african vultures optimization algorithms (AVOA) etc. were used in the literature^16–32^ to solve the optimization problems of MG. A GA has been used in the papers^16^ and^17^ for the energy trading strategy of the MG considering uncertain quantities.

In Ref.^16^, the energy trading strategy was developed for optimal scheduling of conventional generators, energy storage systems, and grid power exchanges. In Ref.^17^, the objective is cost minimization, including the installation cost of battery storage, solar modules, and the operational cost of diesel generators. The goal is subjected to equality constraints like active and reactive power balance and inequality constraints to ensure stability. A modified genetic algorithm has been used in article^18^ to share the power generation among the various DERs optimally. The results show that the modified GA gives better results than the GA.

PSO-based optimization algorithms have been developed in article^19,21,22,33^ for the ELD problems with multiple thermal units, energy storage devices, etc. Some of these works include the impact of large-scale EV integration along with the numerous constraints and load uncertainty. These studies show that the results obtained by the PSO algorithm are much better than those obtained by the GA. However, in article^24–26,34^ researchers have analyzed the performance of variants of PSO named “improved, coordinated aggregation-based particle swarm optimization (ICA-PSO)” algorithm and “PSO with BA parameter inspired acceleration coefficients (MHPSO-BAAC)” to solve the ELD problem with valve point loading for all combinations of RES-based power plants. These algorithms proved that the PSO variants performed better than basic PSO.

In papers^27,35^, another meta-heuristic-based Grey Wolf Optimization algorithm has been developed to solve the economic operation of the microgrid system, the sizing optimization of BESS, etc. The results obtained by GWO have been compared with the results of other meta-heuristic algorithms like GA, PSO, ABC, etc. to show the effectiveness of GWO. A fuzzy PID control based modified slime mould algorithm (MSMA) is developed for optimal battery management system in article^30^. In this article, the tuning of fuzzy PID controller is performed to accommodate the uncertainties of the automatic voltage variation and power management. An African vultures optimization algorithm (AVOA) has been developed in article^31^ for the optimization of a novel two-degree of freedom PID (2DOFPID) controller to emulate the virtual inertia and damping into the Microgrid. The performance of the proposed controller has been compared with the other conventional controller to show its effectiveness. The developed methodlogy^31^ has also been validated on OPAL-RT real time environmental simulator. A slime mold meta-heuristic optimization algorithm for the operation management of Microgrids considering Demand Response Program (DRP) is presented in article^32^. The obtained results show that the developed slime mold optimization algorithm performs better than PSO and Genetic Algorithms.

**Figure 1:**
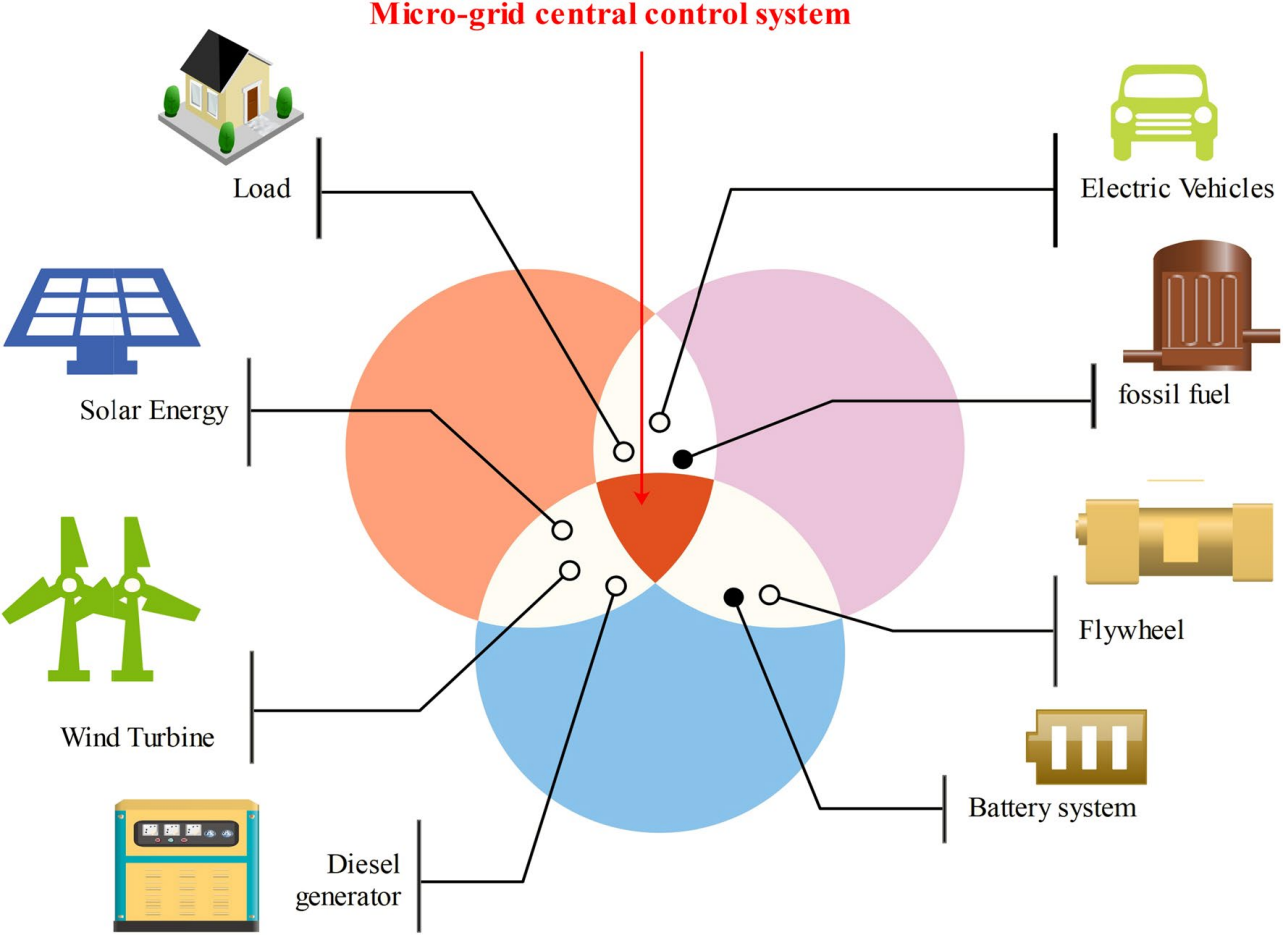
A fundamental architecture of Microgrid.

From the above-discussed literature, the key limitations of the work have been identified and presented as follows: (i) Few studies consider the deterministic approach, and others use the stochastic process; however, computational tractability is an issue. (ii) The scheduling under uncertainty is addressed by a reduced number . of scenarios of load, PV, wind, etc., which makes the system unrealistic. (iii). Only a few pieces of literature have discussed the priority factor-based cost components and the static penalty associated with constraints and limit violations; it needs further investigation.

The main contribution of this work is listed below:

- To tackle the volatile and intermittent nature of PV, wind, and load, maximum scenarios are considered to represent the real system.
- Economic scheduling in both grid-connected and islanded modes uses the concept of load and power curtailment with the help of the GWO algorithm, considering the entire day of system data.
- A combination of the stochastic nature of resources, unpredictable loads, and the heuristic approach to solving the problem.
- An optimal scheduling methodology for MG considering uncertain parameters is proposed along with the existence of an energy storage system.

The remaining paper is organised as follows: In Sect. "Optimal operation of microgrid", the optimal operation of MG is discussed. Section "Results and discussion" describes the results and discussion of the proposed methodology, whereas Sect. "Conclusion" gives the conclusion part of the work.

## Optimal operation of microgrid

The microgrid can be operated in two modes, grid-connected or stand-alone. The fundamental steps of the proposed optimal scheduling strategy of the microgrid in both modes are given:

1. Component modeling
2. Scenario generation and reduction of uncertain parameters
3. Problem formulation
4. Implementation of optimization techniques

### Component modelling

The basic structure of a grid-connected microgrid is shown in Fig. 1, which considers controllable generations, PV generations, wind generations, and energy storage systems.

#### PV system

Sun is the ultimate source of solar energy. The solar irradiance received from the sun can be converted into DC power with the help of solar cells (basic semiconductors), which is further converted into AC power with the help of inverters. The output power ($P_{pv}$) of the PV module depends upon the effective global irradiance, the area of the module, the energy conversion efficiency of the solar module, and the temperature. It is given as,

1 $$\begin{aligned} P_{pv}= G_{eff} \eta _{g}A_g \times [1-0.005(T_c-25)] \end{aligned}$$

where $$G_{eff}$$ is the effective global irradiance $$\eta _{g} $$ is the conversion efficiency of the generator, $$A_g$$ is the active surface area of the module, $$ T_c$$ is the temperature. Generally, the hourly irradiance is modeled by using the Beta distribution function^36^ and is given as,

2 $$\begin{aligned} F_b(G)= {\left\{ \begin{array}{ll} \frac{\Gamma (a+b)}{\Gamma a+\Gamma b} \times G^{(a-1)} \times (1-G)^{b-1} &{} 0 \le G \le 1 \\ &{} a \ge 0,b \ge 0 \\ 0 &{} otherwise \end{array}\right. } \end{aligned}$$

The value of the parameters *a* and *b* is calculated with the help of mean value *u* and standard deviation *n* as,

3 $$\begin{aligned} a= & {} \frac{u \times b}{1-u} \end{aligned}$$

4 $$\begin{aligned} b= & {} (1-u) \times \frac{u(1+u)}{n^2}-1 \end{aligned}$$

#### Wind system

The kinetic energy of wind can be converted into electricity with the help of the wind turbine, however the speed of the wind is intermittent. Therefore, the wind velocity (*u*) is modeled by using the Weibull probability distribution function and is given as^36^,

5 $$\begin{aligned} pdf(u)=\frac{h}{e} \times \left( \frac{u}{e}\right) ^{h-1} \times e^{-(\frac{u}{e})^{h}} \end{aligned}$$

The output power $P_{W}(u)$ of the wind turbine can be modeled in terms of the wind velocity (*u*) as,

6 $$\begin{aligned} P_{W}(u)= {\left\{ \begin{array}{ll} P_{W}^{rated} (u-u_{in})/(u_r-u_{in}) &{} u_{in}\le u \le u_r \\ P_{W}^{rated} &{} u_{in}\le u \le u \\ 0 &{} otherwise \end{array}\right. } \end{aligned}$$

where $P_{W}^{rated}$ (MW) is the rated power output of the wind turbine, *u* (m/s) is the maximum wind speed up to which generation is possible, $u_{in}$ (m/s) is the cut in speed at which wind turbine generates, $u_{r}$ (m/s) is the average wind speed.

#### Controllable distributed generator (CDG)

It is one of the important components of the microgrid to supply the base demand and increase the system’s reliability.

The cost associated with conventional generators is the fuel cost and is modeled as,

7 $$\begin{aligned} FC_i=a \times P_i^2+b \times P_i+c_i \end{aligned}$$

where $P_i$ is the output power, *a*, *b*, and *c* are the fuel consumption curve parameters for any CDG whose units are $\$ /Kw^2h$, $\$ /Kwh$ and $\$$ respectively.

Another significant cost associated with CDGs is the emission cost. It is the penalty for polluting the environment and can be calculated using^37^.

8 $$\begin{aligned} K_i \times  ( m_i \times P_i^2+n_i \times P_i+o_i) \end{aligned}$$

Where $K_i$ is the penalty term for pollution, whose unit is $\$/Kg$. $m_i, n_i$ and $o_i$ are the emission coefficients, whose units are $Kg/Kw^2h$, *Kg*/*Kwh* and *Kg* respectively.

#### Battery energy storage system

It is a device used to store energy. It takes energy from various sources, uses it when required by the loads, and helps balance generation and loads. The battery’s power output can be positive or negative depending on the discharging or charging mode.

This is determined by calculating the net energy and state of charge of the battery^38^.

When the load is greater than a generation, the battery will get discharged,

9 $$\begin{aligned} P_t^{dish} =min(SOC-SOC_{min}, P_{load}-P_{gen}) \end{aligned}$$

similarly, when we have sufficient generation, and the battery is not fully charged, it will be charging,

10 $$\begin{aligned} P_t^{Char} =max(SOC_{max}-SOC, P_{gen}-P_{load}) \end{aligned}$$

### Scenario generation and reduction of uncertain parameters

There is uncertainty associated with renewable generation because of the intermittent nature of wind and solar irradiance. It can be forecasted based on the previous data, but there are some errors. These errors can be modeled using MCS^39^.

#### Scenario generation

To generate the scenarios, there is a need for the forecasted value and error associated with the various stochastic quantities like wind speed (to calculate the wind power) and global irradiance (to calculate PV output) over the entire scheduling horizon^36^.

Then, for each hour, the value of the stochastic quantity is equal to the sum of the forecasted value for that hour, and the error is generated randomly with the help of historical data^40,41^. The same procedure is followed for the load scenario generation as it also keeps changing from time to time and is uncertain.

#### Scenario reduction

Since there are many scenarios for this proposed optimization problem, a proper reduction method must be used to decrease the number of generated techniques so that the solution will become tractable. Clustering is a classic machine learning and computational geometry issue. In this work, we have used the K-mean clustering algorithm. The k-means method is one of the popular clustering methods (unsupervised) where the aim is to reduce the distance between the points of the same cluster^42^.

### Problem formulation

The main objective of microgrid operators is to minimize the overall operating cost of the microgrid by the maximum utilization of renewable energy. The operating cost function of the microgrid is as follows,

11 $$\begin{aligned} \ Min\sum _{s\in S} \pi _s(\alpha  \times J_1 +\beta  \times J_2+\gamma  \times J_3+\delta  \times J_4+\zeta  \times J_5)+\sum _{j=1}^{l+m} \lambda _j \times g_j, \end{aligned}$$

where

12 $$\begin{aligned} J_1= & {} \sum _{t\in T} \sum _{i\in N_i} [C_i^{CDG}(P_{i,t,s}^{CDG})+C_{i,t,s}^{SU}+C_{i,t,s}^{SD}], \end{aligned}$$

13 $$\begin{aligned} J_2= & {} \sum _{t\in T} \sum _{i\in N_i}[m_i(P_i^2)+n_i(P_i)+o_i] \times K_i ,\end{aligned}$$

14 $$\begin{aligned} J_3= & {} \sum _{t\in T} [\rho _{t,s}^{RT,Buy}P_{t,s}^{RT,Buy}-\rho _{t,s}^{RT,Sell}P_{t,s}^{RT,Sell}], \end{aligned}$$

15 $$\begin{aligned} J_4= & {} \sum _{t\in T} [\rho _{t,s}^{Char} \times P_{t,s}^{Char}-\rho _{t,s}^{dish} \times P_{t,s}^{dish}], \end{aligned}$$

16 $$\begin{aligned} J_5= & {} \sum _{t\in T} L_{j,t,s}^{Shed}\lambda _{j,t}^{Voll}], \end{aligned}$$

In Eq. (11), $\pi _s$ is the probability of each scenario, and $\sum _{s\in S} \pi _s=1$, where *S* is the total number of scenarios. The $\alpha ,\beta ,\gamma ,\delta$, and $\zeta$ are the priority factors that are changed for different cases to get the optimal solution. $J_1, J_2, J_3, J_4$, and $J_5$ are the costs associated with CDGs, emission costs, the cost associated with power exchange between microgrid and utility, battery costs, and the value of load loss, respectively. $\sum _{j=1}^{l+m} \lambda _j \times g_j$ represents the penalty term, where $\lambda _j$ is the penalty factor of the $j^{th}$ constraint and $g_j$ represents the $j^{th}$ constraint function that is being violated. *l* and *m* are the equality and inequality constraints, respectively. $C_i^{CDG}$ is the operating cost, and $P_{i,t,s}^{CDG}$ is the power output at time *t* for scenario *s* of the $i^{th}$ conventional generator, whose units are $\$/KWh$ and *Kw*, respectively. $C_{i,t,s}^{SU}$ and $C_{i,t,s}^{SD}$ are the start-up and shut-down costs. $m_i, n_i$, and $o_i$ are the emission cost coefficients of the CDGs. $\rho _{t,s}^{RT,Buy}$ and $\rho _{t,s}^{RT,Sell}$ are the buying and selling prices of electricity with the utility grid, respectively. $P_{t,s}^{RT,Buy}$ and $P_{t,s}^{RT,Sell}$ are the amounts of energy bought and sold to the utility grid, respectively. $P_{t,s}^{Char}$ and $P_{t,s}^{Dischar}$ are the charging and discharging energies of the battery. $\rho _{t,s}^{Char}$ and $\rho _{t,s}^{Dischar}$ are the charging and discharging costs associated with the battery.

The objective function given in (11) is subjected to multiple constraints, among which the power balance is the most important and is given by (17).

17 $$\begin{aligned} P_{PV}+P_W+P_{grid}+P_{CDG}+P_{Lshed}=P_{Load}, \end{aligned}$$

where $$P_{grid}=P_{Buy}-P_{Sell}$$.

Other inequality constraints subjected to the (11) are as follows,

18 $$\begin{aligned}&P_{CDG,Min}\le P_{CDG} \le P_{CDG,Max}, \end{aligned}$$

19 $$\begin{aligned}&P_{PV,Min}\le P_{PV} \le P_{PV,Max}, \end{aligned}$$

20 $$\begin{aligned}&P_{W,Min}\le P_{W} \le P_{W,Max}, \end{aligned}$$

21 $$\begin{aligned}&SOC_{Min}\le SOC \le SOC_{Max}, \end{aligned}$$

$$P_{CDG}$$ is output of CDG and $$P_{CDG,Min}$$ and $$P_{CDG,Max}$$ are the minimum and maximum limits of the power output of CDG. $$P_{PV}$$ is the output of the PV panel, and it has a minimum limit of $$ P_{PV,Min}$$ and a maximum limit of $$P_{PV,Max}$$. $$ P_{W} $$ is the wind output with $$P_{W,Min}$$ and $$P_{W,Max}$$ as the minimum and maximum limit, respectively. State of charge (SOC) is the indicator of battery energy, and it should also be in between minimum $$SOC_{Min}$$ and maximum limit $$SOC_{Max}$$.

### Optimization technique: Grey Wolf optimization GWO

GWO is a population-based metaheuristic algorithm proposed by Mirjaliali et al. in 2014. The social hierarchy and hunting mechanism of grey wolves inspire this algorithm. They belong to the Canidae family, and their scientific name is Canis lupus. Grey wolves are social animals and live together in a group called packs. Each pack consists of 6-12 wolves divided into four categories $\alpha _G$, $\beta _G$, $\delta _G$, and $\omega _G$. The first level leads the pack to decisions such as hunting, sleeping location, etc. They need not be the strongest, but they are best at pack management. The second member of the hierarchy is $\beta _G$. These are the supporting wolves that aid the leader in decision-making. Play the role of discipliner and advisor for the pack. It provides feedback to the alpha and guarantees that all other wolves obey the command. In the absence of $\alpha _G$, they will be leading the team. Sentinels, scouts, hunters, and caretakers form the next hierarchy. Scouts monitor the territory’s boundary and warn the pack in case of danger. Sentinels guarantee safety for the other members of the pack. Hunters and caretakers hunt prey and take care of the pack’s ill and wounded members. Rest all are the $\delta _G$, wolves.

The hunting mechanism of wolves is as follows:

- *Encircling prey*: During the hunt, grey wolves encircle prey, whose position is ($\textbf{X}_{ p}$)
- *Hunting*: $\textbf{A}_{ k}$ and $\textbf{C}_{k}$ are coefficient vectors, here encircling and hunting is done through $\textbf{D}_{k}$ & $\textbf{X}_{new}$, respectively along with new population are computed where, $\textbf{rd}$ is a random number between 0 and 1, and $\textbf{a}_{gwo}$ changes linearly from 2 to 0 with each iteration. The entire process is described below
  22 $$\begin{aligned} \textbf{A}_{ k}= & {} 2\cdot \textbf{a}_{gwo}\cdot \textbf{rd}-\textbf{a}_{gwo}, \end{aligned}$$
  23 $$\begin{aligned} \textbf{C}_{ k}= & {} 2\cdot \textbf{rd}, \end{aligned}$$
  24 $$\begin{aligned} \textbf{D}_{ k}= & {} \Vert \textbf{C}_{ k}\cdot \textbf{X}_{ p}- \textbf{X}_{k}\Vert, \end{aligned}$$
  25 $$\begin{aligned} \mathbf {X'}_{ k}= & {} \textbf{X}_{ k}-\textbf{A}_{ k}\cdot \textbf{D}_{ k}, \end{aligned}$$
  26 $$\begin{aligned} \textbf{X}_{new}= & {} \frac{\sum _{k=1}^{3}\mathbf {X'}_{ k}}{3}, \end{aligned}$$
- *Attacking the prey*: Once the prey stops moving, wolves attack the prey.
- *Exploration*: Grey wolves mainly seek according to the alpha, beta, and delta positions. They disperse from each other to hunt for prey and converge to attack prey. We use random values larger than 1 or less than -1 to mathematically describe divergence to force the search agent to diverge from the prey.

Another feature of GWO that encourages exploration is $\textbf{C}$. This vector has random values in the range [0, 2], allowing GWO to behave more randomly during optimization, promoting exploration and avoiding local optima.

**Algorithm 1:**
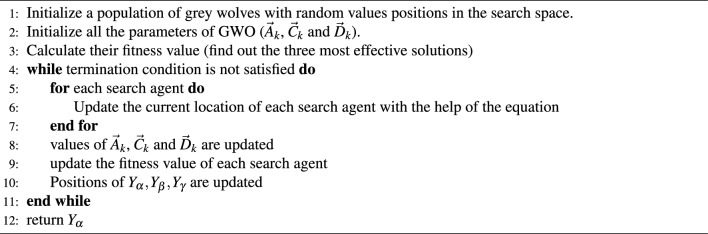
GWO.

*Implementation steps of algorithm*: Implementation of GWO algorithm for the objective function formulated in (11), is illustrated as follows:

- Choose initial parameters and set priority factor $\gamma = 0$ for isolated mode.
- Choose other priority factors $(\alpha _G, \beta _G, \gamma _G$ and $\delta _G)$ for Grid-connected mode.
- Subject to inequality constraints in (18)–(21), minimize the objective function given in (11) using GWO algorithm as described in Algorithm 1.
- Repeat the process for the calculation of $J_1$, $J_2$, $J_3$ and $J_4$ using (12) - (16).
- Stop the procedure once termination criterion meets.

**Figure 2:**
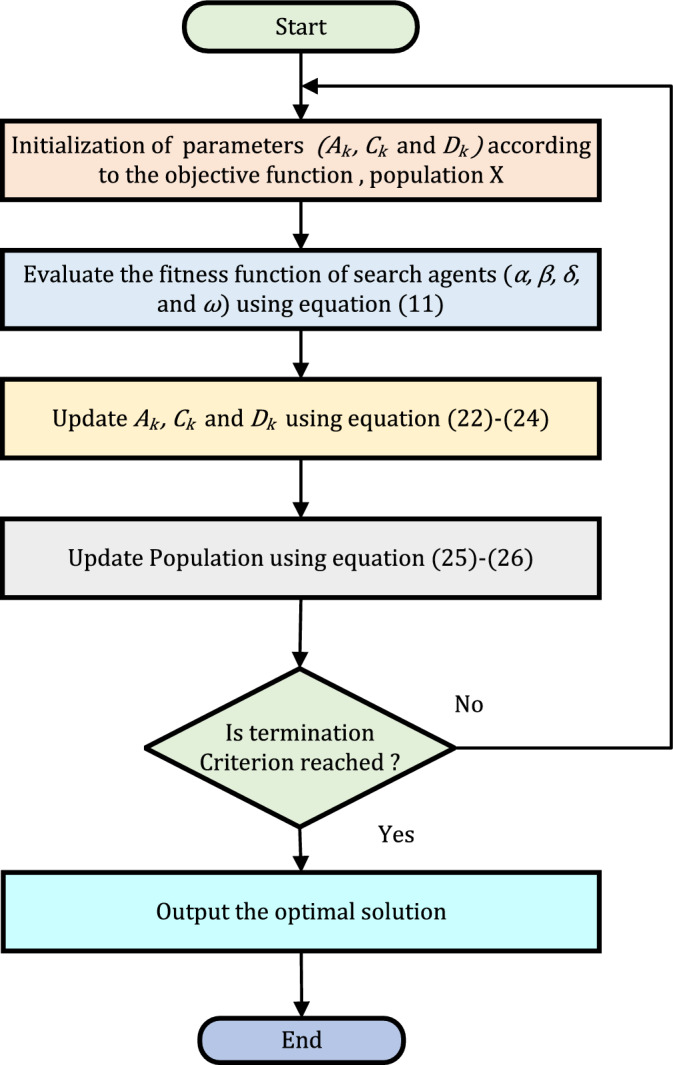
Flowchart of the proposed algorithm.

The flowchart for the proposed algorithm is shown in Fig. 2.

**Figure 3:**
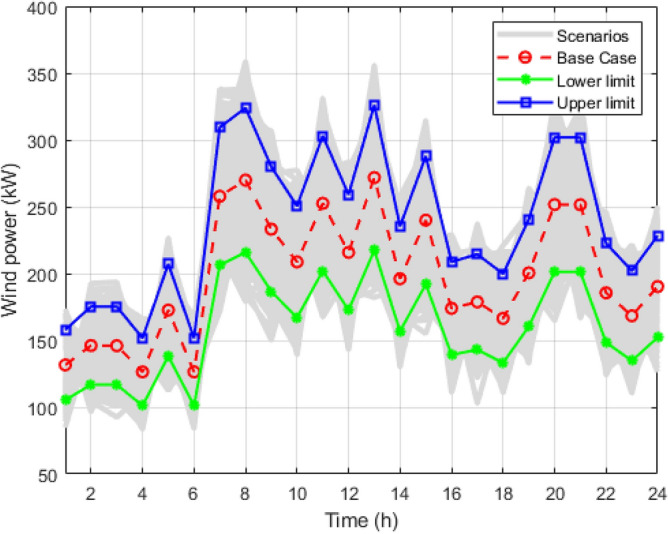
Wind scenarios for 24 hours.

**Figure 4:**
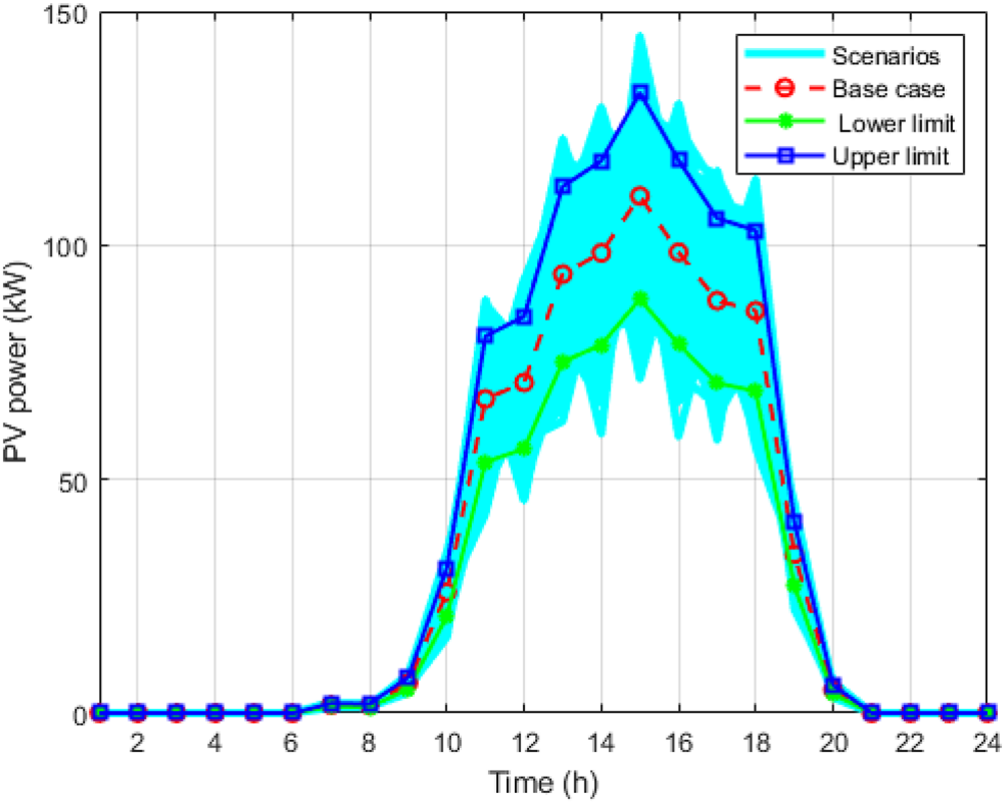
Solar output scenarios for 24 hours.

**Figure 5:**
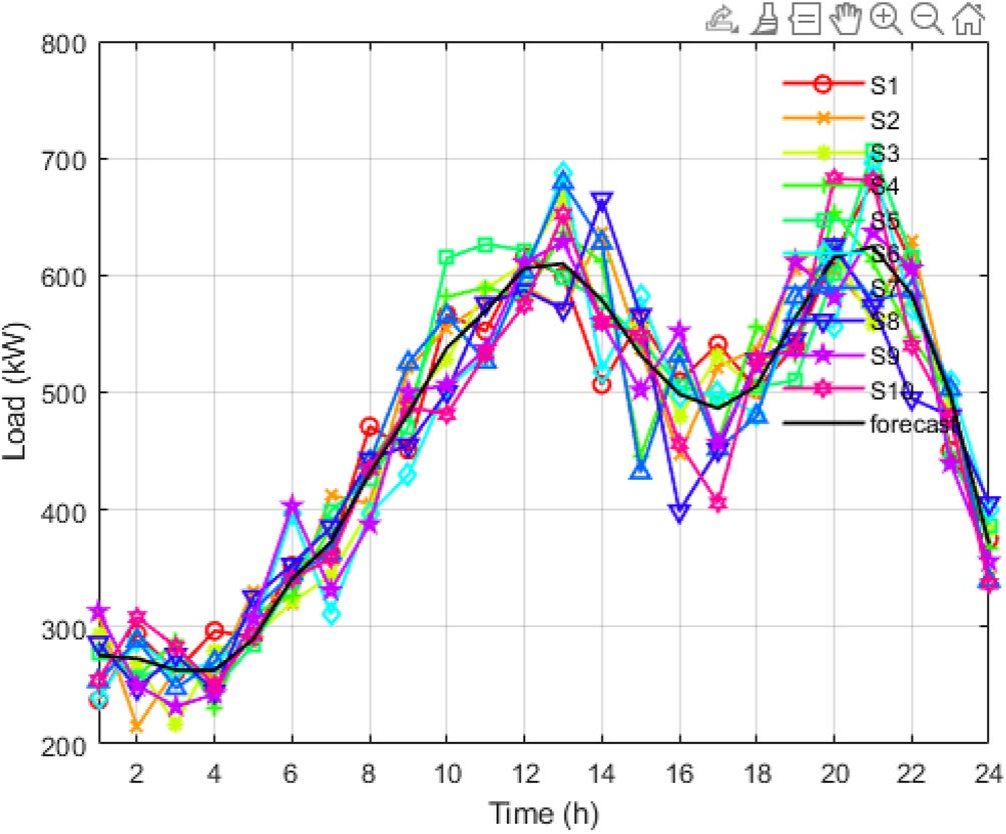
Reduced scenarios of load for 24 hours.

**Figure 6:**
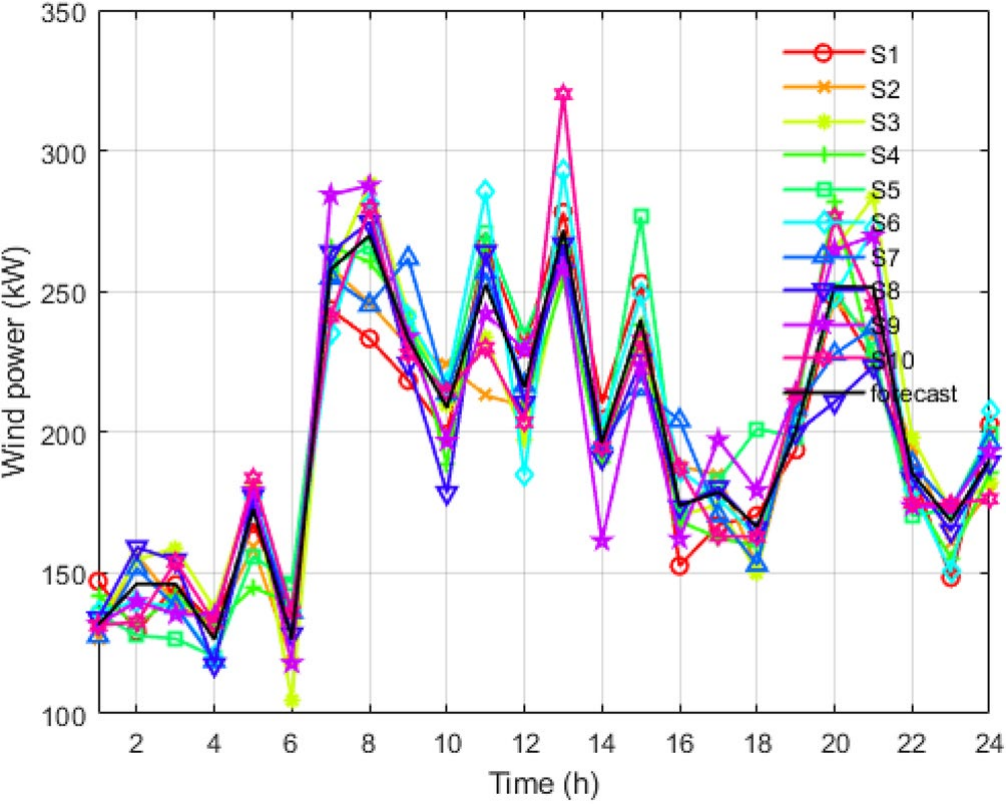
Reduced scenarios of wind output for 24 hours.

**Figure 7:**
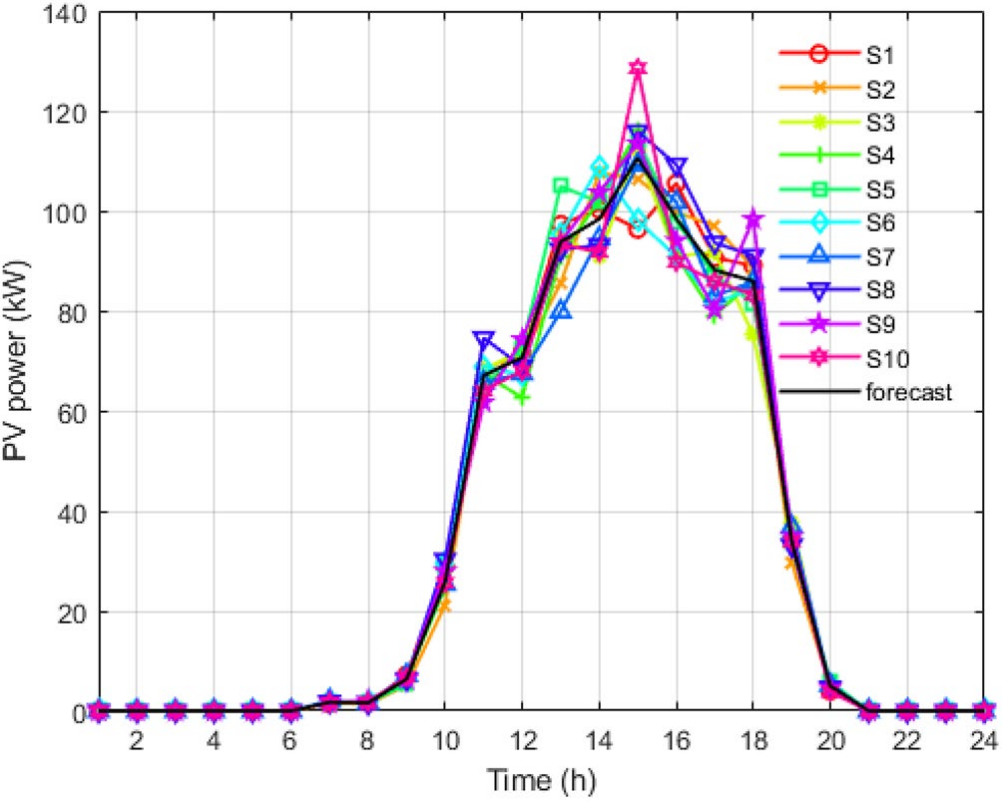
Reduced scenarios of solar output for 24 hours.

## Results and discussion

To validate the proposed methodology, a standard LV Microgrid CIGRE test network is considered. The various data of LV MG CIGRE test system for wind turbine, photovoltaic, battery energy storage system, controllable load etc. are collected from^43^. The GWO algorithm, as discussed in Sect. Optimization technique: Grey Wolf optimization GWO, is implemented in MATLAB software to get the optimal solution of the developed optimization problem, along with the Jaya and PSO algorithms. Each search agent in the optimisation approach has number of variables that keep changing every hour. Cost analysis is carried out to demonstrate the efficiency of GWO and Jaya. At first, the total 2000 scenarios for loads, PV output, and wind output were generated to get all the uncertainties associated with them, as discussed in the Sect. Scenario generation and reduction of uncertain parameters.

**Figure 8:**
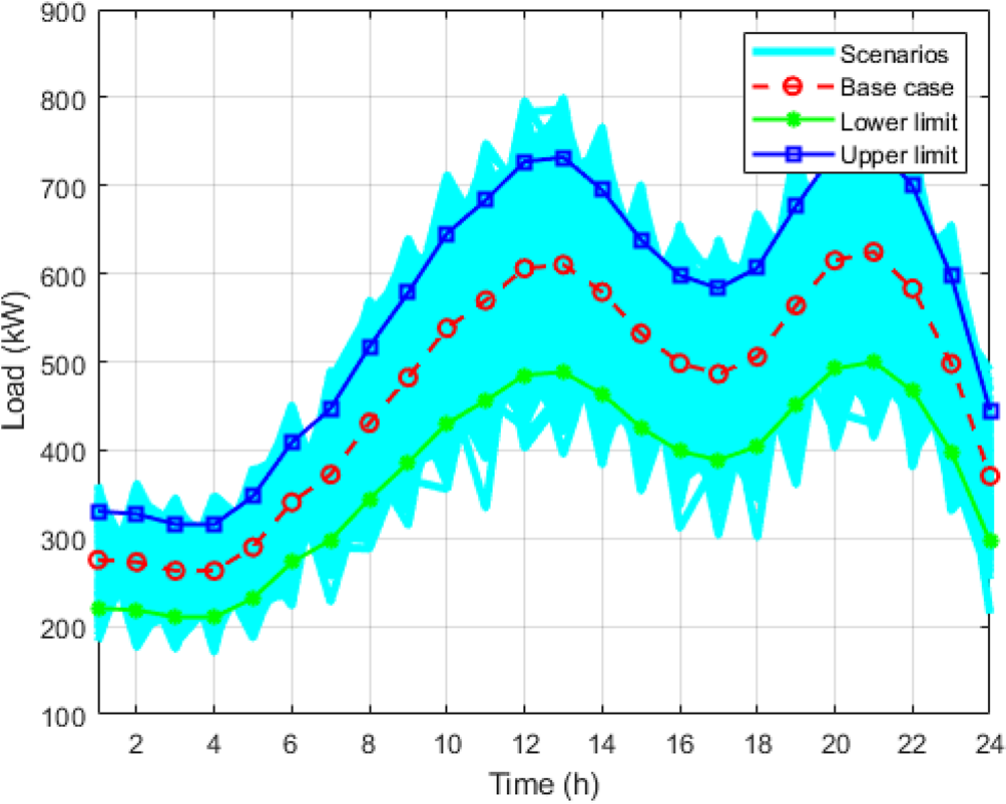
Load scenario for 24 hours.

**Table 1:** Different cases considered for optimization.

	$\alpha$	$\beta$	$\gamma$	$\delta$
Case 1	0.75	0.04	0.1	0.1
Case 2	0.6	0.1	0.2	0.1
Case 3	0.5	0.1	0.25	0.15
Case 4	0.5	0.15	0.2	0.15
Case 5	1	1	1	1

**Figure 9:**
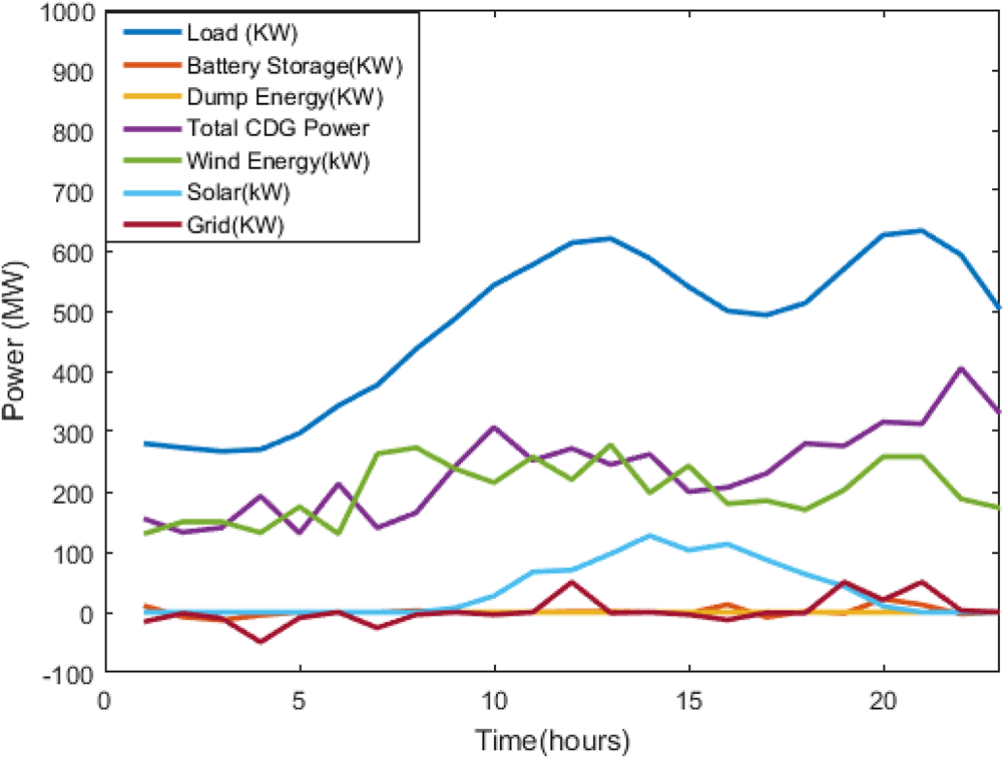
power profiles of the CDGs, renewables, and Grid Supply System considering battery using GWO.

**Figure 10:**
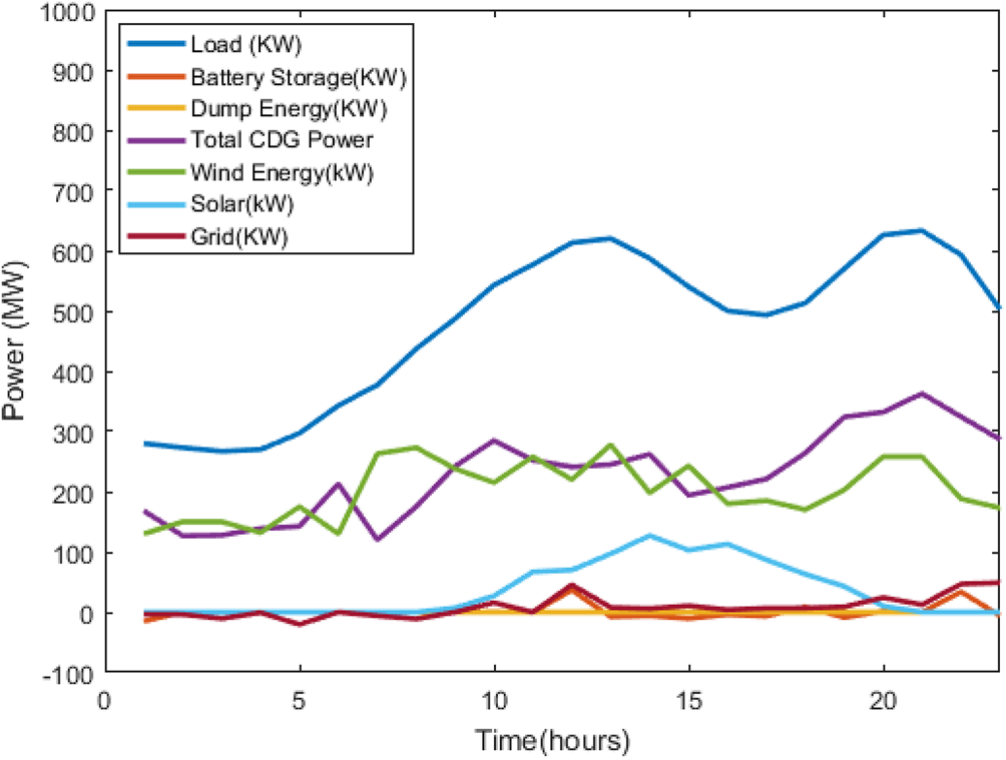
power profiles of the CDGs, renewables, and grid supply system considering battery using Jaya.

**Table 2:** Various cost components for different cases in grid-connected mode.

		Jaya	PSO	GWO
Case-1	J1 ($\$$)	6084	6181.61	6051
	J2 ($\$$)	0.53	0.52	0.526
	J3 ($\$$)	0.74	3.62	2.5
	J4 ($\$$)	2.55	3.25	1.16
Case-2	J1 ($\$$)	4903	4853	4898
	J2 ($\$$)	1.39	1.28	1.33
	J3 ($\$$)	– 2.03	7	– 0.5
	J4 ($\$$)	0	3.7	0.48
Case-3	J1 ($\$$)	4098	4104	4034
	J2 ($\$$)	1.36	1.34	1.327
	J3 ($\$$)	– 1.37	5.55	– 3.48
	J4 ($\$$)	2.219	3.73	3
Case-4	J1 ($\$$)	4113.04	4290	4002
	J2 ($\$$)	14.029	1.9	2
	J3 ($\$$)	– 26.0191	5.2	– 3.8
	J4 ($\$$)	5.759	3.8	3.65
Case-5	J1 ($\$$)	8188	8160	8037
	J2 ($\$$)	14.0229	13	13.57
	J3 ($\$$)	– 26.0191	36	– 24.2
	J4 ($\$$)	6	28	28.59

**Table 3:** Cost comparison using JAYA, PSO, and GWO algorithm in grid-connected mode.

		Jaya	PSO	GWO
Case-1	Best cost ($\$$)	5683	5689	5583
	Worst cost ($\$$)	6435	6530	6312
	Average cost ($\$$)	6088	6189	6051
	Standard deviation ($\$$)	194	270	262
Case-2	Best cost ($\$$)	4358	4490	4549
	Worst cost ($\$$)	5446	5203	5176
	Average cost ($\$$)	4903	4865	4898
	Standard deviation ($\$$)	200	227	216
Case-3	Best cost ($\$$)	3853	3846	3841
	Worst cost ($\$$)	4445	4281	4277
	Average cost ($\$$)	4101	4104	4036
	Standard deviation ($\$$)	210	160	159
Case-4	Best cost ($\$$)	3856	3866	3755
	Worst cost ($\$$)	4449	6133	4278
	Average cost ($\$$)	4112	4290	4004
	Standard deviation ($\$$)	210	659	183
Case-5	Best cost ($\$$)	7026	7716	7616
	Worst cost ($\$$)	8775	8794	8704
	Average cost ($\$$)	8182	8160	8055
	Standard deviation ($\$$)	515	308	391

**Table 4:** Various cost components for different cases in Isolated mode.

		Jaya	PSO	GWO
Case-1	J1 ($\$$)	7619	6428	5702
	J2 ($\$$)	0.53	0.5	0.36
	J3 ($\$$)	24	36	172
	J4 ($\$$)	0.844	0.54	0.344
Case-2	J1 ($\$$)	6579	5030	4685
	J2 ($\$$)	1.15	1.23	0.88
	J3 ($\$$)	77	34	184
	J4 ($\$$)	1.637	1.8	0.3
Case-3	J1 ($\$$)	5839	4116	3972
	J2 ($\$$)	1.26	1.234	0.94
	J3 ($\$$)	67.316	49.22	242
	J4 ($\$$)	3.85	1.9	0.62
Case-4	J1 ($\$$)	5839	4122	3976
	J2 ($\$$)	2	1.87	1.44
	J3 ($\$$)	67	38	242
	J4 ($\$$)	3.08	1.1	0.12
Case-5	J1 ($\$$)	9110	8133	8326
	J2 ($\$$)	12	12	12.14
	J3 ($\$$)	381	3030	407
	J4 ($\$$)	12.45	4.22	4.152

**Table 5:** Cost comparison using JAYA, PSO, and GWO algorithm in isolated mode.

		Jaya	PSO	GWO
Case-1	Best cost ($\$$)	6239	5956	5513
	Worst cost ($\$$)	9397	7301	5874
	Average cost ($\$$)	7188	6428	5690
	Standard deviation ($\$$)	1106	423	122
Case-2	Best cost ($\$$)	4893	4708	4350
	Worst cost ($\$$)	6788	6991	4840
	Average cost ($\$$)	5708	5315	4662
	Standard deviation ($\$$)	612	689	176
Case-3	Best cost ($\$$)	4222	3944	3786
	Worst cost ($\$$)	11116	5359	4176
	Average cost ($\$$)	6193	4302	4050
	Standard deviation ($\$$)	2450	409	116
Case-4	Best cost ($\$$)	4373	3985	3754
	Worst cost ($\$$)	9152	5666	4189
	Average cost ($\$$)	6074	4544	3976
	Standard deviation ($\$$)	1614	525	131
Case-5	Best cost ($\$$)	820	7897	8136
	Worst cost ($\$$)	11608	9104	9106
	Average cost ($\$$)	9730	8560	8691
	Standard deviation ($\$$)	1023	383	300

Figure 8 shows all the strategies generated for load using the MCS for the entire day. The blue line curve shows the upper limit curve of the load profile, the green line shows the lower limit of the load curve, and the red dotted curve is the forecasted load curve. Figure 3 is all the scenario sets for the wind power output with a deviation of 15%. The blue curve is the upper limit of the wind curve for 24 hours; the green colour shows the lower acceptable limit curve for wind scenarios; and the red curve shows the predicted values of the wind but is highly uncertain compared to the load and PV scenarios. Figure 4 shows the multiple techniques for solar output generated with the help of MCS using the base value forecasted (red curve) and the error produced using their probability distribution function. The blue and green lines in the set of scenarios show the upper and lower limits of the solar output with the maximum deviations.

It is reduced into smaller samples using the K-mean clustering algorithms to reduce the computational time and complexity. Figure 5 is the graph obtained after applying the k-mean algorithm to the load scenario graph. The set of two thousand load curves is reduced to the group of ten load curves represented by *S*1, *S*2, *S*3, *S*4, *S*5, *S*6, *S*7, *S*8, *S*9, and *S*10, respectively. In Figs. 6 and 7, the total 2000 of wind power output and solar power output curves are also reduced to a set of 10 curves represented as *S*1 to *S*10 with the help of K-mean clustering, respectively. Data sets of PV, wind, and load are obtained with their associated probabilities for each of the ten scenarios.

### Grid connected mode

The grid can be considered the virtual generator. A microgrid can buy power when there is a deficit and supply power when it has excess renewable generation.

In Table 1, different priority factor terms like $\alpha$, $\beta$, $\gamma$, $\delta$, and $\zeta$ show the relative significance of each cost component in the overall objective function. Based on these priority factors, five cases have been considered.

- Case1: The value of $\alpha$ is taken as .75, it shows the relative significance of fuel cost $J_1$, the value of $\beta$ is .04, it is the weight factor for the emission cost $J_2$, and the values of $\gamma$ and $\delta$ are 0.1 and 0.1, respectively, and these show the weight factor of the cost associated with energy exchanged with grid $(J_3)$ and battery cost $(J_4)$, respectively.
- Case2: The value of $\alpha$ is taken as .6, which is associated with fuel cost $J_1$; the value of $\beta$ is 0.1; it is the weight factor for the emission cost $J_2$; and the values of $\gamma$ and $\delta$ are 0.2 and 0.1, respectively, and these show the weight factors of $J_3$ and $J_4$, respectively.
- Case3: The value of $\alpha$ is reduced to .5, the value of $\beta$ is .1, and the values of $\gamma$ and $\delta$ are 0.25 and 0.15, respectively.
- Case4: The value of $\alpha$ is .5, the value of $\beta$ is .15, and the values of $\gamma$ and $\delta$ are 0.2 and 0.15, respectively.
- Case5: For this case, the values of $\alpha$, $\beta$, $\gamma$, and $\delta$ are assumed to be 1.

In each case, 10 scenarios are taken, with (*PV*1*W*1*L*1) or *S*1 of PV from Fig. 8, *S*1 of wind from Fig. 7, *S*1 of load from Fig. 6 being the first scenario or the first data set, *PV*2*W*2*L*2 (S2 of PV, S2 of wind, S2 of load) being the second scenario (second data set), and so on. For these data sets, optimisation is carried out using the GWO and Jaya algorithms. For each scenario, there is an optimal value, but we are focusing on stochastic optimisation, so to consider the uncertain nature, we need to take the average optimal values of all the scenarios.

From Table 3, GWO gives better results than Jaya for all the cases. The lowest cost for case 4 in the GWO algorithm value is $4004\$$. The standard deviation is less when using GWO and quite high when using Jaya.

The main cost is the operating cost of CDGs, followed by the grid exchange cost, battery cost, and emission cost. The same can be observed from Table 2, when the priority of all the cost terms is equal, the cost is maximum, as in case 5 of all the optimisation methods, but if it is allotted properly, the overall cost reduces. From Table 3, GWO is giving better results than the Jaya algorithm for my problem. Although the difference between the optimal cost obtained from GWO and Jaya is not high for one day, it will significantly affect the cost for longer. Figure 9 shows the load curve, total of CDGs power, wind power output, solar output, exchange with the grid, battery power output, and dump energy (PL-PG) by using the GWO algorithm. Similarly, Fig. 10 shows the load curve, the total of CDGs power, wind power output, solar output, exchange with the grid, battery power output, and the dump using the Jaya algorithm.

### Isolated mode

In this mode, there is no grid to act like a virtual generator, so we will go for load curtailment when we don’t have sufficient generation. This load curtailment is associated with some revenue loss for the operator, which we call VOLL (value of loss load), which is higher than the price of electricity offered to the customer. In isolated mode, the third term of the objective function (exchange with the grid) is not there, and the Lshed term comes into the picture.

- Case1: The value of $\alpha$ is taken as .75, it shows the relative significance of fuel cost $J_1$, the value of $\beta$ is .04, it is the weight factor for the emission cost $J_2$, and the values of $\zeta$ and $\delta$ are 0.1 and 0.1, respectively, and these show the weight factor of the cost associated with load shedding $J_5$ and battery cost $(J_4)$, respectively.
- Case2: The value of $\alpha$ is taken as .6, which is associated with fuel cost $J_1$; the value of $\beta$ is .1; it is the weight factor for the emission cost $J_2$; and the values of $\zeta$ and $\delta$ are 0.2 and 0.1, respectively, and these show the weight factors of $J_5$ and $J_4$, respectively.
- Case3: The value of $\alpha$ is reduced to .5, the value of $\beta$ is .1, and the values of $\zeta$ and $\delta$ are 0.25 and 0.15, respectively.
- Case4: The value of $\alpha$ is .5, the value of $\beta$ is .15, and the values of $\zeta$ and $\delta$ are 0.2 and 0.15, respectively.
- Case5: For this case, the values of $\alpha$, $\beta$, $\zeta$, and $\delta$ are assumed to be 1.

Table 5 observed that the overall cost has increased in isolated mode on the operator side sometimes when GWO optimization is used and for all instances where the Jaya algorithm is used. This is because of load curtailment to balance demand and supply and improve reliability. Load curtailment is associated with incentives given to the customer because of overall cost increases. For the GWO algorithm, the best optimal cost is 3754$ for case 4, and the worst case is 9106$, whereas for Jaya, the best case is case 3 with 4222$ dollars, and the worst case is case 5 with 11608$. The detailed description of different cost components obtained in isolated mode is in Table 4 for PSO, GWO, and Jaya.

## Conclusion

This paper proposes a day-ahead stochastic scheduling problem for the MG with uncertainty. The main aim is to minimise the overall cost of the microgrid, and a scenario-based method is modelled for the uncertain nature of RESs (PV and wind) and load. The economic load dispatch problem has been solved using two popular metaheuristic algorithms, the Grey-Wolf algorithm and Jaya. Jaya and PSO performed equally well compared to GWO. The proposed strategy’s effectiveness in economics and reliability is investigated on a standard benchmark LV microgrid CIGRE test network. Economic load dispatch was performed for both the grid-connected and the islanded microgrid. During isolated mode, the cost was maximised by the Jaya algorithm and a little less by GWO. In grid-connected mode, GWO has obtained the best optimal solution.

## Data Availability

The datasets used and/or analysed during the current study available from the corresponding author on reasonable request.
